# MALE ROLE NORMS AND THE PREVALENCE OF POST-TRAUMATIC STRESS DISORDER SYMPTOMS AMONG POLISH MALE PARAMEDICS

**DOI:** 10.13075/ijomeh.1896.02545

**Published:** 2025

**Authors:** Magdalena Maja Sitko-Dominik, Tomasz Daniel Jakubowski, Eugenia Ewa Mandal

**Affiliations:** University of Silesia in Katowice, Faculty of Social Sciences, Institute of Psychology, Katowice, Poland

**Keywords:** Poland, post-traumatic, social support, stress disorders, masculinity, paramedics

## Abstract

**Objectives:**

Knowledge of the incidence of post-traumatic stress disorder (PTSD) in the rescue profession is crucial for designing psycho-education to minimize the risk of its occurrence and increase the chance of providing effective assistance. The aim of the study was to analyze risk factors that may increase the likelihood of developing PTSD among paramedics.

**Material and Methods:**

The study was conducted on 113 male paramedics. The study used the *Impact of Event Scale – Revised*, the *Male Role Norms Scale* and the *Social Support Scale*.

**Results:**

The results showed that nearly 40% of the respondents suffer from PTSD. The paramedics who met the criteria for PTSD did not differ in their acceptance of male norms or the level of perceived social support from the paramedics who did not develop PTSD symptoms. Multiple regression analyses showed that the number of types of events experienced in the course of professional duties and male cultural norms related to social status were predictors of PTSD. Logistic regression analysis revealed that acceptance of male norm of anti-femininity increased the likelihood of developing PTSD.

**Conclusions:**

The results showed that men who performed stereotypically male professions could be less likely to seek specialist help in PTSD situations because such behavior does not fit the role of a strong, assertive man in social perception.

## Highlights

- The results have shown that nearly 40% of the respondents suffer from post-traumatic stress disorder (PTSD).
- Adhering to anti-femininity norms increases the likelihood of developing PTSD.
- The male cultural norms related to social status are predictors of PTSD.

## INTRODUCTION

There are still few studies concerning the relationship between social and interpersonal variables and post-traumatic stress disorder (PTSD) [1]. The present study draws on the previous research and focuses on the significance of the traditional understanding of the male social role as a potential risk of PTSD development in male paramedics experiencing traumatic events.

The profession of a paramedic is consistent with the stereotype of masculinity as paramedics are perceived as heroes, while the archetype of the paramedic is embodied by service, care and self-control [2]. Additionally, this image is also maintained by the mass media [3]. Paramedics themselves seem to share this opinion, as they believe that they are perceived by society as “saviours” and this role corresponds to hegemonic masculinity [4].

In addition, the profession of paramedic allows achieving high social status, which is in line with masculine norms [5,6]. It is shown that professional prestige is a stronger determinant of competence than its objective indicators [7]. In Poland, the profession of a paramedic is much more often performed by men than women. The profession is highly respected by the public, as evidenced by the fact that paramedics are ranked second in terms of socially respected professions directly after firefighters [8,9]. However, despite their high social prestige, their status is considered low when referring to objective indicators in the form of remuneration, promotion opportunities or social power [10].

Although paramedics are perceived as heroes, it does not always correspond with their professional experience [2]. While performing their duties, paramedics experience high levels of stress because they are responsible for the health and lives of the affected individuals. They frequently experience helplessness while providing them with assistance, or are confronted with death. Additionally, significant stress and challenges are related to communication with the relatives of the affected individuals, as the close ones experience pain and despair, and hence they may also question the competence of paramedics and treat them with disrespect [11,12]. Furthermore, media reports on incidents of paramedics falling victim to physical assault get more frequent; these assaults show that they are at risk of experiencing violence from patients or their relatives [13–15]. Taking the above into consideration, paramedics are exposed to potentially traumatic factors during their professional duties, which can increase the risk of developing PTSD. It is estimated that 5.4–20% of paramedics are affected by PTSD [16–19], while in Poland it affects 28–41.9% of them [12,20,21].

According to a review of studies by Berger et al. [22], paramedics experience PTSD more often than other emergency service representatives, such as firefighters and police officers. In addition, the population of paramedics is characterised by not only higher levels of PTSD but also depression, anxiety, fatigue and insomnia compared to the general population. They also experience chronic stress, sleep disorders and poorer mental well-being and are more likely to abuse alcohol and suffer from obesity [16,17,23,24], which may be associated with the performance of this profession by men [25,26].

Paramedics are characterised by high levels of denial and relatively low levels of empathy, which may be considered adaptive coping in a stressful work environment [18]. In the short-term perspective, maintaining distance from the affected individuals and their families can be effective. However, in the-long term perspective, it can adversely affect interpersonal relationships of paramedics [27].

Potential problems in interpersonal relationships and in communicating one's emotions may directly make it difficult for paramedics to obtain support, as research conducted among the other first responders (among whom are firefighters, police officers and paramedics) group–police officers – shows that the likelihood of developing PTSD increases in those who have difficulty in expressing emotions [28,29]. Furthermore, understanding masculinity in a traditional way (as well as conforming to the traditional norms pertaining to it) may be responsible for the severity of alexithymia [30,31]. According to the Polish study, alexithymia is associated with PTSD in paramedics [32].

Men who perform male-dominated professions are more likely to conform to traditional male norms, while the workplace culture may also be responsible for the above state of affairs, thus affecting physical and mental health, as men may choose not to talk about problems they experience for fear of being seen as “unmanly,” and because independence is important to them [33,34]. Bearing it in mind, despite some potential benefits of talking to their fellow paramedics about what they experience and declaring the wish to get support from co-workers, friends or family members, they may choose not to do so under real circumstances [4,35]. Furthermore, in this professional group, reluctance to seek and obtain help, including the one provided by professionals is reported [12]. Such reluctance is found particularly in men, as they show a lower propensity to seek help compared to women who also perform male-dominated occupations [36].

Perceived rather than received support is more important for paramedics to maintain their mental health [37] and supportive work relationships can protect against trauma [38]. However, paramedics experience low levels of support [17] and perceive the work environment to be highly demanding, characterised by low control and being poorly supportive [39], while the support deficit may be responsible for the increased risk of developing PTSD [12]. Paramedics who are prepared to cope with potentially traumatic events experienced in the course of their professional duties and who receive psychological support present with less severe PTSD symptoms and a higher sense of coherence than paramedics for whom these services were not available [40]. Additionally, young men who enter the paramedic profession increasingly refuse to accept the suppression of emotions characteristic of the profession and traditional masculinity [4].

Gender stereotypes are simplistic judgments shared by society that are related to the characteristics and behaviours of women and men acquired through the socialisation process that takes place within a culture [41–43]. They have both descriptive and prescriptive functions, hence different expectations towards men and women [44]. However, regardless of age, men have more restrictive prescriptive stereotypes [45]. According to the structure of gender stereotypes within the framework of the stereotype of masculinity, 4 components can be distinguished. They are related to mental and physical characteristics, as well as social and professional roles [46,47]. Also, studies conducted on the Polish population found that such components are stable [48].

Cislaghi and Heise [42] define gender norms as social norms that determine acceptable and desirable behaviours for men and women. Therefore, men are expected to be strong, independent, tough and self-controlled. They are also expected to achieve high social status and avoid behaviours that are stereotypically perceived as feminine [5,6]. Understanding masculinity in a traditional way is considered more often a risk factor than a protective factor, as it can reduce the likelihood of seeking help when faced with difficulties [49–51].

Conforming to traditional masculine roles by men can also negatively affect their perceptions of available social support and make them less likely to use constructive coping strategies or seek help for fear of being perceived as weak or unmanly [52,53]. It is indicated that whether men seek help depends on how such help is perceived by them. However, they may hold negative attitudes towards it and be less likely to seek it because such behaviour is perceived as oppositional to masculinity [54–56]. Recent research findings also show that the relationship between traditional masculinity and help-seeking is mediated by attitudes towards help-seeking and partly by self-stigma, and partly moderated by the severity of depression [57]. The literature does not provide information on the relationship between traditional masculinity and the risk of developing psychopathology among paramedics, in the case of which, according to other studies [12,20,21], PTSD is prevalent in that group.

### The present study

As far as the authors know, there is no Polish research directly investigating the relationship between male role norms, PTSD and social support in the population of paramedics. To fill the gap, the authors aim to explore the significance of the traditional understanding of the male social role as a potential risk of PTSD development in male paramedics experiencing traumatic events.

Male paramedics are exposed to potentially traumatic events in the course of their professional duties and behaviours consistent with the traditional masculine role may result in the development of maladaptive behaviours that will limit the ability to obtain support and increase the likelihood of developing PTSD.

Additionally, the above issue may be intensified by culture. Polish researchers describe Polish culture as hard to define or feminine [58,59]. However, based on Hofstede's cultural dimensions theory, Polish culture is considered masculine in which the roles of men and women are distinct. As a result, men can show greater masculine traits [43,60,61].

Identification with cultural masculinity may induce men to conform to the social expectations directed towards them [58,62]. It is noted that the extent to which men respect traditional masculine norms depends on culture [63], and the study results of Valved et al. [64] in the Polish (masculine culture) and Norwegian (feminine culture) groups show that Poles prescribe more agency to men than Norwegians. Poles are also more affected when given feedback that they have feminine gender knowledge (their masculinity is threatened).

In relation to the research aim and the employed theoretical framework, the following research questions were formulated:

– What are the potential associations between compliance with traditional masculinity norms, the level of perceived social support and the intensity of PTSD symptoms in male paramedics?
– Are there any differences in compliance with traditional masculinity norms and the level of perceived social support between male paramedics who meet the criteria for PTSD and those who do not?
– Is compliance with traditional masculinity norms, the level of perceived social support, age, education, length of service, length of relationship, the number of types of potentially traumatic events and the frequency of potentially traumatic events the predictors of PTSD?

The following hypotheses were formulated:

– The level of compliance with traditional masculinity norms is negatively associated with the level of perceived social support, and positively associated with the intensity of PTSD symptoms among male paramedics.
– The paramedics who meet the criteria for PTSD are more compliant with traditional masculinity norms and report lower levels of social support than the paramedics who do not meet the criteria for PTSD.
– Compliance with traditional masculinity norms, age, length of service, number of types of potentially traumatic events and frequency of potentially traumatic events are positive predictors of PTSD, while education and length of relationship are negative ones.

## MATERIAL AND METHODS

### Participants and procedures

The study was a part of the project pertaining to PTSD among first responders (police officers, firefighters and paramedics). The protocol of the current study is available online [65].

The research was conducted online using Google Forms in September 1, 2020 – September 30, 2021. There was no direct contact with the participants due to the risk of COVID-19 transmission. The study design was approved by the Ethics Committee of the University of Silesia in Katowice for research involving human subjects (No. KEUS.68/01.2021). The written informed consent was obtained from all participants.

The study participants were recruited using the snowball method. To obtain a representative sample with respect to a particular centre, the researchers with students (who acted as assistants) invited paramedics to participate in the study by sending invitations to selected hospitals or posting them on forums for paramedics. Participation in the study was not associated with any benefits, including financial profits, for the subjects and research assistants. The male paramedics who gave their informed consent to participate in the study were asked to complete 3 questionnaires and provide information about their age education, length of service and relationship length.

### Measures

#### The *Impact of Event Scale – Revised* (IES-R)

The Polish adaptation of Daniel Weiss and Charles Marmar's [66] questionnaire by Juczyński and Ogińska-Bulik [67]. The measure consists of 22 items scored on a 5 – point Likert scale (from 0 – “definitely not” to 4 – “definitely yes”) divided into 3 sub-scales: intrusions (e.g., “When I recalled the event, the emotions returned”), hyper arousal (e.g., “Recalling this event made me sweat and I had breathing problems, dizziness, and my heart fluttered”) and avoidance (e.g., “I tried to avoid talking about the event”). The higher is the score of a given sub-scale, the higher is the intensity of PTSD symptomatology. The reliability of the whole scale in the current study was α = 0.95; the sub-scale reliability was α = 0.92, α = 0.84 and α = 0.84 for intrusion, hyperarousal and avoidance, respectively.

According to Creamer et al. [68], the diagnosis of PTSD is possible when the mean result of the whole scale is >1.5. However, Juczyński and Ogińska-Bulik [67] proposed a more restrictive approach, i.e., the researchers suspect that the individual meets the criteria for PTSD when mean values of all the 3 sub-scales (intrusion, hyperarousal and avoidance) are ≥1.5. The method might decrease the chance of overestimating the prevalence of PTSD among the respondents.

#### The *Male Role Norms Scale*

The *Male Role Norms Scale* by Thompson and Pleck [6] was translated into Polish by Jakubowski and Sitko-Dominik. The measure consists of 26 items scored on a 7 – point Likert scale (from “I definitely don't agree” to “I definitely agree”) in 3 sub-scales: anti-femininity norms (e.g., “If I learnt about a man that is a hairdresser or a cook, I would wonder how masculine he is”), toughness (e.g., “I like men who are totally self-confident”) and social status (e.g., “Success at work must be the main aim in the man's life”). The higher is the score of a given sub-scale, the higher is the level of compliance with a given masculinity norm. The reliability of the scale in the study was α = 0.93, while the reliability of the social status, toughness and anti-femininity norms sub-scales was α = 0.90, α = 0.83 and α = 0.71, respectively.

#### The *Social Support Scale*

The *Social Support Scale* by Skarżyńska [69] consists of 6 items (e.g., “In important matters I can count on my friends' help”; “I am surrounded by many people who are close to me”) scored on a 5-point Likert scale (from “I definitely don't agree” to “I definitely agree”). The higher is the score, the higher is the level of perceived social support. The reliability of the scale in the current study was α = 0.75. The participants were requested to provide information on their age, gender, education, length of service, marital status and length of the relationship. They were asked to tick off what they experience during their service: being injured, seeing dead bodies, death of a fellow paramedic, experiencing a life threat/assault. The participants were questioned how often they experienced such potentially distressing situations (from “there were no such events” to “more than a dozen times”). The list was adapted from the study conducted by Dudek [70].

### Statistical analysis

The IES-R total score and the 3 symptom criteria of PTSD (avoidance, hyperarousal, intrusion) were dependent variables in the study. The independent variables included social status norms, toughness norms, anti-femininity norms, social support, education, age, length of service, relationship length, number of types of events and frequency of events. Statistica software package v. 13.1. [71] was used to compute descriptive statistics, intergroup differences, correlations, logistic regression and univariate regression. The Spearman's rank correlation was used to assess potential associations between compliance with masculinity norms (social status, toughness and anti-femininity norms), social support and PTSD symptomatology (intrusion, avoidance, hyperarousal and IES-R total score) due to non-normal distribution of most of the variables (except for hyperarousal).

Next, the Mann-Whitney U test was applied to evaluate potential differences in social support and compliance with toughness, anti-femininity and social status norms between paramedics who met the criteria for PTSD (the positive screen for the PTSD group, according to Juczyński and Ogińska-Bulik's [67] restrictive approach) and those who did not (the negative screen for the PTSD group).

To assess potential predictors of PTSD symptomatology (male role norms, social support and sociodemographic data), the authors conducted univariate regression. Statistical significance was set at p < 0.05.

## RESULTS

The study group consisted of 113 male paramedics working in Poland (age mean [M] and standard deviation [SD] 32.16±7.74). The mean length of service was approx. 9 years (M±SD 8.77±7.53). Most of the respondents had a higher education (N = 91, 80.54%), secondary education was declared by 19 men (16.81%), and vocational education was indicated by 3 men (2.65%). Among the study participants, 48 men were married (42.48%), 39 subjects were in informal relationships (34.51%), 22 were single (19.47%), while 4 were divorced (3.54%). The mean relationship length was approx. 6 years (M±SD 6.31±6.86).

The frequency of experiencing traumatic events showed that most paramedics indicated seeing dead bodies and human remains as a traumatic event in the course of their professional duties (N = 105, 92.92%), and the least the death of 1 of the paramedics (N = 1, 0.88%). As regards the events that evoked the greatest shock that could not be forgotten, most participants indicated the sight of dead bodies and the remains of children (N = 51, 45.14%). The frequency of experiencing traumatic events in which paramedics participated and other medical variables are given in Tables 1 and 2, respectively.

**Table 1.** The frequency of experiencing traumatic events among Polish paramedics, Poland, 2020–2021

Traumatic event	Participants [n (%)]	
	total*	the event evoked shock and became unforgettable
1. You saw dead bodies and human remains.	105 (92.92)	30 (26.56)
2. You were in a hostile and aggressive crowd.	85 (75.22)	7 (6.19)
3. You saw dead bodies and the remains of children.	65 (57.52)	51 (45.14)
4. Other paramedics were attacked and injured.	48 (42.48)	3 (2.65)
5. You were attacked and your life was directly threatened.	47 (41.59)	8 (7.08)
6. Other paramedics were attacked and their lives were directly threatened.	46 (40.71)	4 (3.54)
7. You were attacked and injured.	41 (36.28)	9 (7.96)
8. You injured someone.	9 (7.96)	0 (0)
9. One of the paramedics died.	1 (0.88)	1 (0.88)

* The percentages do not add up because paramedics could indicate all the events they had participated in when they were on duty.

**Table 2.** Basic descriptive statistics for the variables included in the study of Polish paramedics, Poland, 2020–2021

Variable	M	SD	Skewness	Kurtosis
The IES-R total score	1.46	0.93	0.38	−0.53
Intrusion	1.41	1.06	0.54	−0.63
Hyperarousal	3.16	1.16	0.26	0.21
Avoidance	1.60	1.02	0.10	−1.00
Social support	3.79	0.74	−0.21	−0.72
Social status norms	3.87	6.27	−0.20	−0.51
Toughness norms	3.33	1.31	0.07	−0.74
Anti-femininity norms	2.76	1.03	0.62	−0.11

IES-R – *Impact of Event Scale – Revised*.

The Spearman correlation analysis was conducted (Table 3) to test whether there are potential associations between compliance with traditional masculinity norms, the level of perceived social support and the intensity of PTSD symptoms in male paramedics. The correlation analyses showed that the IES-R total score correlated positively with social status norms (r = 0.263). For intrusion, the analysis showed that it co-existed with social status norms (r = 0.291). In turn, hyperarousal correlated positively with norms for avoidance of feminine behaviour (r = 0.670), social status (r = 0.794) and toughness (r = 0.896). In addition, avoidance correlated positively with social status norms (r = 0.226).

**Table 3.** Spearman's rank correlation analysis results included in the study of Polish paramedics, Poland, 2020–2021

Variable	Spearman's rank correlation coefficient							
	1	2	3	4	5	6	7	8
1. Social support	1.000	−0.097	−0.010	−0.033	−0.099	−0.012	0.009	−0.047
2. Intrusion		1.000	0.150	0.753***	0.947***	0.291**	0.130	0.143
3. Hyperarousal			1.000	0.178	0.154	0.794***	0.896***	0.670***
4. Avoidance				1.000	0.892***	0.226*	0.163	0.133
5. The IES-R total score					1.000	0.263**	0.134	0.145
6. Social status norms						1.000	0.672***	0.551***
7. Toughness norms							1.000	0.602***
8. Anti-femininity norms								1.000

IES-R – *Impact of Event Scale – Revised*.

* p < 0.05;

** p < 0.01;

*** p < 0.001.

The authors assessed the occurrence of PTSD among paramedics following the approach proposed by Juczyński and Ogińska-Bulik [61] to check whether there are differences in compliance with traditional masculinity norms and the level of perceived social support between male paramedics who met the criteria for PTSD and those who did not meet such criteria. The results showed that 44 (39.84%) paramedics met the criteria for PTSD.

On the basis of the above findings, the authors divided the study group into 2 subgroups:

– paramedics who met the criteria for PTSD (N = 44, 39.84%),
– paramedics who did not meet the criteria for PTSD (N = 69, 61.06%).

Next, the authors conducted an analysis of the differences between the subgroups in terms of compliance with traditional masculinity norms and the level of perceived social support between male paramedics. No statistically significant differences were found between the subgroups.

The authors conducted a series of univariate regressions to identify predictors of PTSD symptomatology. In the light of the results, the number of types of events experienced in the course of professional duties (β = 0.24, p < 0.001) and social status norms (β = 0.31, p < 0.001) were statistically significant predictors of the intensity of PTSD symptoms. The model based on the above predictors accounted for 14% of the variance in PTSD symptom intensity. The results of the regression analysis are given in Table 4.

**Table 4.** Results of univariate regression analyses for the dependent variable (intensity of post-traumatic stress disorder [PTSD] symptoms) based on the *Impact of Event Scale-Revised* included in the study of Polish paramedics, Poland, 2020–2021

Predictor	β	SEE β	T	R^2^	R^2^_adj_	F	df	p
Education*	0.03	0.09	7.13	0.001	0.01	0.08	1.111	0.777
Number of types of potentially traumatic events	0.24	0.09	2.61	0.06	0.05	6.79	1.111	<0.001
Social status norms	0.31	0.09	2.39	0.10	0.09	12.01	1.111	<0.001

* Dichotomised variable: 0 – secondary education, 1 – higher education.

Additionally, the authors conducted a series of logistic regressions for the dichotomised dependent variable – probable PTSD (1) and no PTSD (0). The analyses showed that compliance with anti-femininity norms (OR = 1.90, p = 0.034) statistically significantly predicted whether PTSD could occur. Therefore, the authors can conclude that the higher is the compliance with anti-femininity norms, the greater is the likelihood of developing PTSD among paramedics. The results of the regression analysis are given in Table 5.

**Table 5.** Results of logistic regression analyses for the dichotomised variable – probably post-traumatic stress disorder (PTSD) (1) and no PTSD (0) included in the study of Polish paramedics, Poland, 2020–2021

Predictor	OR (95% CI)	p
Age	0.99 (0.88–1.11)	0.827
Education*	0.72 (0.28–1.83)	0.486
Length of service	1.02 (0.90–1.16)	0.723
Length of the relationship	0.96 (0.87–1.07)	0.493
Number of types of potentially traumatic events	1.09 (0.88–1.36)	0.424
Frequency of potentially traumatic events	1.19 (0.76–1.85)	0.453
Social support	1.33 (0.79–2.34)	0.292
Social status norms	0.72 (0.45–1.16)	0.173
Toughness norms	0.86 (0.53–1.41)	0.547
Anti-femininity norms	1.90 (1.05–3.44)	0.034

* Dichotomised variable: 0 – secondary education, 1 – higher education.

## DISCUSSION

The main aim of the study was to describe the factors contributing to the development of PTSD among male paramedics. The obtained results, indicating that almost 40% of the male respondents met the criteria for PTSD, are in line with other studies conducted in Poland [12,20,21]. It is worth noting that the prevalence of PTSD in Poland is much higher than in the studies conducted among paramedics in Western Europe [16–19,72,73]; similarly, in the case of the prevalence in the general population [74]. According to Rzeszutek et al. [74], these disparities might result from decreased access to professional help due to the Polish Healthcare system underfunding as well as the potentially low socioeconomic status of paramedics that bars them from the access to specialists in the private sector. Yet another explanation of these differences, according to Rzeszutek et al. [74] and Szumiał [75], might lie in the utilization of convenience sample as well as time of the study, which was conducted during the onset of the COVID-19 pandemic.

The analyses showed that PTSD symptoms correlated with compliance with the masculinity norms. The authors found a positive correlation between the IES-R total score and compliance with social status norms. It is likely that paramedics are not sufficiently satisfied, considering their involvement in work, remuneration and the status of their profession. The tendency to seek higher earnings and material status is associated with extra work, overwork, and greater involvement in the paramedic job, which results in a higher number of PTSD symptoms. It may also indicate a greater tendency to neglect one's own needs and not to seek help when faced with difficulties [52,76].

The relationship between avoidance and social status norms may be explained as follows: paramedics experience internalised social pressure to achieve high social status [6]. Therefore, they avoid discussions on traumatic events and reaching out for professional help, which prevents them from working through the trauma [52,77,78]. The stereotype of masculinity, which was adopted by paramedics during primary and secondary socialisation that occurs as part of masculine culture, may also be responsible for it [43,60,61,79].

Hidden messages about socio-cultural categories of masculinity and femininity are present in every area of life [80]. The portrayal of men as resourceful and calm, and the focus on the professional status they obtain are evident as early as in preschool education when the image of the father is presented [81]. The relationship between intrusion and compliance with social status norms, which was presented in this study, can also be explained in a similar way.

As regards hyperarousal, which is another PTSD component, the study showed that it co-occurred with social status norms, toughness norms and anti-femininity norms. Bearing it in mind, the authors can conclude that paramedics may choose to use maladaptive coping strategies to deal with stress and thus consume alcohol or take drugs, as such behaviours correspond with a traditional perception of masculinity [52,79,82,83].

On the basis of the regression analyses, the authors found that the frequency of potentially traumatic events and masculinity norms associated with social status were independent predictors of the development of PTSD. In an attempt to explain the results, the authors have come to the conclusion that experiencing many types of traumatic events may make it difficult for paramedics to cope with stressful situations, which may lead to an increase in the intensity of PTSD symptoms. Due to the specificity of their profession, paramedics are at higher risk of being involved in potentially traumatic events and may experience exhaustion due to work overload or burnout [84–88].

Compliance with anti-femininity norms significantly increase the likelihood of developing PTSD. Paramedics may refrain from seeking help and applying adaptive coping strategies for fear of being perceived as “weak” and thus “unmanly” [6,52,53,82,89,90]. The above can be due to the fact that their profession is in line with the stereotype of masculinity since, according to Milner et al. [36], men in male-dominated occupations are less likely to seek specialised medical and psychological help.

The authors did not find a relationship between compliance with masculinity norms, social support and PTSD symptomatology. Paramedics are highly respected in Polish society [8,9]; this makes it possible for them to experience social approval and positive reactions of the social environment. Social acclaim constitutes a protective factor in PTSD development [91,92]. However, afraid of losing the social status, they may have a negative attitude to emotion expression and experience difficulties with identifying emotions [93], because such behavior is in opposition to the traditional conception of masculinity, thus they may suppress their emotions.

It is said that men suppress emotions more often than women. The strategy is in line with the traditional conception of masculinity [52,83,94]. In addition, it may be more frequently employed by paramedics, which is determined by culture. It is indicated that both the use of reappraisal and suppression strategies may be explained by means of Hofstede's dimensions of culture [95]. Suppression at the individual level may make establishing close relations difficult and decrease the possibility of getting support [96,97], however it may also be related to positive consequences at the group level, because, as Matsumoto et al. [95], show, a lower suppression level is related to greater happiness and to maladjustment.

Paramedics may get support, however it may be superficial. In other words, they may be surrounded by their loved ones, but they do not talk to them about their experiences, afraid of being perceived as “unmanly,” or not to burden them with their problems [4,35]. It may also be a consequence of distancing from the patients; this may translate into the paramedics' interpersonal relations, which are less close than the average [27].

The research [98,99] shows that healthcare does not respond to paramedics' needs. Furthermore, getting professional support may be difficult for them both due to problems with talking about their emotions and the specificity of their profession. Paramedics work shifts, as a consequence they may experience problems with regular therapy participating, while chargeable healthcare may not be available to them due to their relatively low income.

### Limitations of the study

There are several important limitations to the current study. First, a tendency among people with PTSD to avoid situations which remind them of traumatic events might have resulted in lower participation in the study of paramedics with significant PTSD symptomatology, which in turn could have possibly contributed to underestimating its rates as well as the statistical significance of its relationships with other analysed variables.

Second, conducting an online study is a convenient way to acquire data from the respondents. It might encourage individuals who use the Internet to participate and to reveal personal information on delicate matters [100]. It might also be influenced by report and recall bias.

Third, the cross-sectional nature of the study might have hindered the identification of associations between the analysed variables, which might have been significant in the long run. This might also be influenced by the Polish attitude towards masculinity, which is traditionalistic [48,61,76] and might result in underreporting PTSD symptomatology and overestimating compliance with male role norms.

Fourth, the relatively small sample size might have impacted all the above mentioned.

## CONCLUSIONS

The aim of the study was to explore potential risk and protective factors contributing to the development of PTSD symptomatology in the face of experiencing adverse events. Almost 40% of the respondents were found suffering from probable PTSD. If it represents the occurrence of PTSD in the general population of male paramedics, this calls for immediate action.

As the study revealed, the main action should consist of psychoeducation about PTSD symptoms, its relations with traditionalist male role norms, especially in the context of help-seeking behaviours, often diminished or prevented by the norms [3,4,52,82]. This might be applied in cognitive-behavioural interventions which target, among others, maladaptive beliefs (in this case, those related to gender norms).

This is further reinforced by the fact that, as indicated by this study, anti-femininity norms significantly increase the odds of developing PTSD. In addition, the general population of first responders (i.e., paramedics, police officers and firefighters) is underserved by mental healthcare [98,99].
